# BrAPI v2: real-world applications for data integration and collaboration in the breeding and genetics community

**DOI:** 10.1093/database/baaf048

**Published:** 2025-09-24

**Authors:** Peter Selby, Rafael Abbeloos, Anne-Francoise Adam-Blondon, Francisco J Agosto-Pérez, Michael Alaux, Isabelle Alic, Khaled Al-Shamaa, Johan Steven Aparicio, Jan Erik Backlund, Aldrin Batac, Sebastian Beier, Gabriel Besombes, Alice Boizet, Matthijs Brouwer, Terry Casstevens, Arnaud Charleroy, Keo Corak, Chaney Courtney, Mariano Crimi, Gouripriya Davuluri, Kauê de Sousa, Jeremy Destin, Stijn Dhondt, Ajay Dhungana, Bert Droesbeke, Manuel Feser, Mirella Flores-Gonzalez, Valentin Guignon, Corina Habito, Asis Hallab, Jenna Hershberger, Puthick Hok, Amanda M Hulse-Kemp, Lynn Carol Johnson, Sook Jung, Paul Kersey, Andrzej Kilian, Patrick König, Suman Kumar, Josh Lamos-Sweeney, Laszlo Lang, Matthias Lange, Marie-Angélique Laporte, Taein Lee, Erwan Le Floch, Francisco López, Brandon Madriz, Dorrie Main, Marco Marsella, Maud Marty, Célia Michotey, Zachary Miller, Iain Milne, Lukas A Mueller, Moses Nderitu, Pascal Neveu, Nick Palladino, Tim Parsons, Cyril Pommier, Jean-François Rami, Sebastian Raubach, Trevor Rife, Kelly Robbins, Mathieu Rouard, Joseph Ruff, Guilhem Sempéré, Romil Mayank Shah, Paul Shaw, Becky Smith, Nahuel Soldevilla, Anne Tireau, Clarysabel Tovar, Grzegorz Uszynski, Vivian Bass Vega, Stephan Weise, Shawn C Yarnes, The BrAPI Consortium

**Affiliations:** Plant Breeding and Genetics Section, School of Integrative Plant Science, Cornell University, Ithaca, NY 14853, USA; VIB Agro-incubator, Vlaams Instituut voor Biotechnologie (VIB), 9850 Nevele, Belgium; IFB-core, Institut Français de Bioinformatique (IFB), CNRS, INSERM, INRAE, CEA, 91057 Evry, France; Université Paris-Saclay, INRAE, BioinfOmics, URGI, 78026, Versailles, France; Plant Breeding and Genetics Section, School of Integrative Plant Science, Cornell University, Ithaca, NY 14853, USA; Université Paris-Saclay, INRAE, BioinfOmics, URGI, 78026, Versailles, France; MISTEA, University of Montpellier, INRAE, Institut Agro, 34000 Montpellier, France; International Center for Agricultural Research in the Dry Areas (ICARDA), Dalia Bldg, Bashir El Kassar Street, VFPM+GJW, Bayrut, Lebanon; International Center for Tropical Agriculture (CIAT), Cali, Valle del Cauca, Colombia; Integrated Breeding Platform, 56237 El Batán, Texcoco, México, México; Integrated Breeding Platform, 56237 El Batán, Texcoco, México, México; Leafnode LLC, 56237 El Batán, Texcoco, México, México; Institute of Bio- and Geosciences (IBG-4: Bioinformatics), CEPLAS, Forschungszentrum Jülich GmbH, Wilhelm Johnen Straße, 52428 Jülich, Germany; Bioeconomy Science Center (BioSC), Forschungszentrum Jülich GmbH, 52428 Jülich, Germany; MISTEA, University of Montpellier, INRAE, Institut Agro, 34000 Montpellier, France; CIRAD, UMR AGAP Institut, 34980, Montpellier, France; AGAP Institut, CIRAD, INRAE, Institut Agro, Université de Montpellier, Montpellier, 34980, France; Wageningen University and Research, Droevendaalsesteeg, Wageningen, 2, 6708 PB, Netherlands; Buckler Lab and Institute for Genomic Diversity, Cornell University, Ithaca, NY 14853, USA; MISTEA, University of Montpellier, INRAE, Institut Agro, 34000 Montpellier, France; USDA-ARS Genomics and Bioinformatics Research Unit, 5601 Sunnyside Avenue, Beltsville, MD 20705, USA; Department of Plant and Environmental Sciences, Clemson University, 16 N Clemson Ave, Clemson, SC 29631, USA; Integrated Breeding Platform, 56237 El Batán, Texcoco, México, México; Leibniz Institute of Plant Genetics and Crop Plant Research (IPK), 06466 Seeland, Germany; Bioversity International, Parc Scientifique Agropolis II, 34397 Montpellier, France; Université Paris-Saclay, INRAE, BioinfOmics, URGI, 78026, Versailles, France; VIB Agro-incubator, Vlaams Instituut voor Biotechnologie (VIB), 9850 Nevele, Belgium; College of Agriculture, Louisiana State University (LSU), 106 Martin D. Woodin Hall Baton Rouge, LA 70803, USA; VIB Data Core, Vlaams Instituut voor Biotechnologie (VIB), 9052 Ghent, Belgium; Leibniz Institute of Plant Genetics and Crop Plant Research (IPK), 06466 Seeland, Germany; Graduate School DILS, Bielefeld Institute for Bioinformatics Infrastructure (BIBI), Postfach 10 01 31, 33501 Bielefeld, Germany; The Boyce Thompson Institute, Ithaca, NY 14853, USA; Bioversity International, Parc Scientifique Agropolis II, 34397 Montpellier, France; Integrated Breeding Platform, 56237 El Batán, Texcoco, México, México; Institute of Bio- and Geosciences (IBG-4: Bioinformatics), CEPLAS, Forschungszentrum Jülich GmbH, Wilhelm Johnen Straße, 52428 Jülich, Germany; Bingen Technical University of Applied Sciences, Berlinstraße, Bingen am Rhein, 109, 55411, Germany; Department of Plant and Environmental Sciences, Clemson University, 16 N Clemson Ave, Clemson, SC 29631, USA; Diversity Arrays Technology (DArT), University of Canberra Kirinari Street, Bruce, ACT, 2617, Australia; USDA-ARS Genomics and Bioinformatics Research Unit, 5601 Sunnyside Avenue, Beltsville, MD 20705, USA; Buckler Lab and Institute for Genomic Diversity, Cornell University, Ithaca, NY 14853, USA; Department of Horticulture, Washington State University, Pullman, WA 99164, USA; Royal Botanic Gardens, Kew, Richmond, London, TW9 3AE, England, UK; Diversity Arrays Technology (DArT), University of Canberra Kirinari Street, Bruce, ACT, 2617, Australia; Leibniz Institute of Plant Genetics and Crop Plant Research (IPK), 06466 Seeland, Germany; Leibniz Institute of Plant Genetics and Crop Plant Research (IPK), 06466 Seeland, Germany; Plant Breeding and Genetics Section, School of Integrative Plant Science, Cornell University, Ithaca, NY 14853, USA; Bingen Technical University of Applied Sciences, Berlinstraße, Bingen am Rhein, 109, 55411, Germany; Leibniz Institute of Plant Genetics and Crop Plant Research (IPK), 06466 Seeland, Germany; Bioversity International, Parc Scientifique Agropolis II, 34397 Montpellier, France; Department of Horticulture, Washington State University, Pullman, WA 99164, USA; Université Paris-Saclay, INRAE, BioinfOmics, URGI, 78026, Versailles, France; International Treaty on Plant Genetic Resources for Food and Agriculture, FAO, Viale delle Terme di Caracalla, 00153 Rome, Italy; MrBot Software Solutions, Cartago, 30501, Costa Rica; Department of Horticulture, Washington State University, Pullman, WA 99164, USA; International Treaty on Plant Genetic Resources for Food and Agriculture, FAO, Viale delle Terme di Caracalla, 00153 Rome, Italy; Université Paris-Saclay, INRAE, BioinfOmics, URGI, 78026, Versailles, France; Université Paris-Saclay, INRAE, BioinfOmics, URGI, 78026, Versailles, France; Buckler Lab and Institute for Genomic Diversity, Cornell University, Ithaca, NY 14853, USA; Department of Information and Computational Sciences, The James Hutton Institute, Invergowrie, Dundee, Scotland; The Boyce Thompson Institute, Ithaca, NY 14853, USA; SEQART AFRICA, Old Naivasha road, 00100, Nairobi, Kenya; MISTEA, University of Montpellier, INRAE, Institut Agro, 34000 Montpellier, France; Breeding Insight, Cornell University, Ithaca, NY 14853, USA; Breeding Insight, Cornell University, Ithaca, NY 14853, USA; Université Paris-Saclay, INRAE, BioinfOmics, URGI, 78026, Versailles, France; CIRAD, UMR AGAP Institut, 34980, Montpellier, France; AGAP Institut, CIRAD, INRAE, Institut Agro, Université de Montpellier, Montpellier, 34980, France; Department of Information and Computational Sciences, The James Hutton Institute, Invergowrie, Dundee, Scotland; Department of Plant and Environmental Sciences, Clemson University, 16 N Clemson Ave, Clemson, SC 29631, USA; Plant Breeding and Genetics Section, School of Integrative Plant Science, Cornell University, Ithaca, NY 14853, USA; Bioversity International, Parc Scientifique Agropolis II, 34397 Montpellier, France; Royal Botanic Gardens, Kew, Richmond, London, TW9 3AE, England, UK; CIRAD, UMR INTERTRYP, Montpellier, France INTERTRYP, Univ Montpellier, CIRAD, IRD, French Institute of Bioinformatics (IFB), Montpellier, 34980, France; South Green Bioinformatics Platform, Bioversity, CIRAD, INRAE, IRD, 34980 Montpellier, France; College of Agriculture and Life Sciences, North Carolina State University, Raleigh, NC 27695, USA; Department of Information and Computational Sciences, The James Hutton Institute, Invergowrie, Dundee, Scotland; Department of Information and Computational Sciences, The James Hutton Institute, Invergowrie, Dundee, Scotland; Integrated Breeding Platform, 56237 El Batán, Texcoco, México, México; Leafnode LLC, 56237 El Batán, Texcoco, México, México; MISTEA, University of Montpellier, INRAE, Institut Agro, 34000 Montpellier, France; Integrated Breeding Platform, 56237 El Batán, Texcoco, México, México; Leafnode LLC, 56237 El Batán, Texcoco, México, México; Diversity Arrays Technology (DArT), University of Canberra Kirinari Street, Bruce, ACT, 2617, Australia; Bingen Technical University of Applied Sciences, Berlinstraße, Bingen am Rhein, 109, 55411, Germany; Leibniz Institute of Plant Genetics and Crop Plant Research (IPK), 06466 Seeland, Germany; Breeding Insight, Cornell University, Ithaca, NY 14853, USA; The BrAPI Consortium, Cornell University, Ithaca, NY 14853, USA

## Abstract

Population growth and the impacts of climate change are placing increasing pressure on global agriculture and breeding programmes. Recent advancements in phenotyping techniques, genotyping technologies, and predictive modelling are accelerating genetic gains in breeding programmes, helping researchers and breeders develop improved crops more efficiently. However, these advancements have also led to an overwhelming torrent of fragmented data, creating significant challenges in data integration and management. To address this issue, the Breeding Application Programming Interface (BrAPI) project was established as a standardized data model for breeding data. BrAPI is an international, community-driven effort that facilitates interoperability among databases and tools, improving the sharing and interpretation of breeding-related data. This open-source standard is software-agnostic and can be used by anyone interested in breeding, phenotyping, germplasm, genotyping, and agronomy data management. This manuscript provides an overview of the BrAPI project, highlighting the significant progress made in the development of the data standard and the expansion of its community. It also presents a showcase of the wide variety of BrAPI-compatible tools that have been built to enhance breeding and research activities, demonstrating how the project is advancing agricultural innovation and data management practices.

## Introduction

Breeding programmes aim to deliver improved lines or cultivars, the most fundamental input for farming, and are thus foundational for maintaining a productive agricultural system amidst the pressing challenges of climate change. Breeding efforts are time- and resource-intensive, with progress dependent on efficient programme logistics and accurate selection decisions. While breeding programmes can benefit from modern and emerging breeding techniques like genomic selection, machine learning, and high-throughput phenotyping, the successful implementation of these methods depends on the ability to efficiently collect, manage, and analyse large volumes of carefully curated genomic and phenomic data [1]. Extracting actionable knowledge from these complex datasets is time-consuming, often prohibiting the adoption of new methods, especially by under-resourced breeding programme. To facilitate the collection, management, and analysis of these datasets, it is essential to transition to digital tools. Historically, independent applications were designed to address specific problems, but in many cases, this led to separate software solutions for each breeding programme task and created data silos.

The Breeding Application Programming Interface (BrAPI) is a standardized, web service application programming interface (API) specification for breeding and related agricultural data [2]. Since the project’s inception in 2014, BrAPI has become an essential part of the digital infrastructure for breeding, providing a domain-specific open data standard tailored to the needs of breeding and genetics projects. BrAPI enables interoperability between breeding software platforms, allowing groups to seamlessly share data and software tools both within and across breeding programmes. It eases the merging of datasets of different types and provides access to shared trait ontologies, phenotypic data, genotypes, seed inventories, and other essential components for collaborative breeding efforts.

Since its first publication in 2019 [2], BrAPI has seen a significant increase in community services, compatible tools, and participating organizations. The community has organized numerous hackathons to evolve the specification, resulting in continuous improvements and enhancements. This report includes a short technical description of the standard and a showcase of the applications, services, and tools available from the BrAPI community. It is the intention of this manuscript to demonstrate the value of BrAPI to the wider scientific community as an effective and efficient means to collaborate and exchange data.

### How it works

An API is a technical connection between two pieces of software. Just as a graphical user interface (GUI) or a command line interface (CLI) allows a human user to interact with a piece of software, an API allows one software application to interact with another. BrAPI is based on the representational state transfer (REST) technical architecture, which describes the stateless transmission of data between applications [3]. Typically, REST-style (or RESTful) web service APIs are implemented using the standard HTTP protocol that most of the modern internet is built upon. These implementations generally use JavaScript Object Notation (JSON) to represent the data being transferred. Both HTTP and JSON are programming language agnostic, very stable, and highly flexible. This means BrAPI can be implemented in almost any piece of software and can solve a wide range of use cases.

Data repositories and service providers that are BrAPI compatible have mapped their internal data structures to the BrAPI standard models, allowing them to share data with the outside world in a standardized format. Similarly, they can accept new data from external sources and automatically map the new data to their existing database. The service providers publish a set of RESTful web service paths, also called ‘endpoints’, for authorized client applications to use to access the services. Client application developers can take advantage of this standardization by building tools and connectors that integrate with all BrAPI-compatible data repositories. Visualization, reporting, analytics, data collection, and quality control tools can be built once and shared with other organizations that follow the standard. This type of BrAPI-compatible, easily sharable tool is often referred to as a BrAPP, meaning BrAPI Application. BrAPPs are simple tools that are entirely reliant on BrAPI for their data requirements, and often fit on a single web page. A single BrAPP can be easily shared and used by many organizations and systems, as long as those organizations have the required BrAPI service endpoints available. As the number of BrAPI-compatible databases, tools, and organizations grows, so does the value of implementing the standard into any given application.

### Project updates

**Figure 1.**
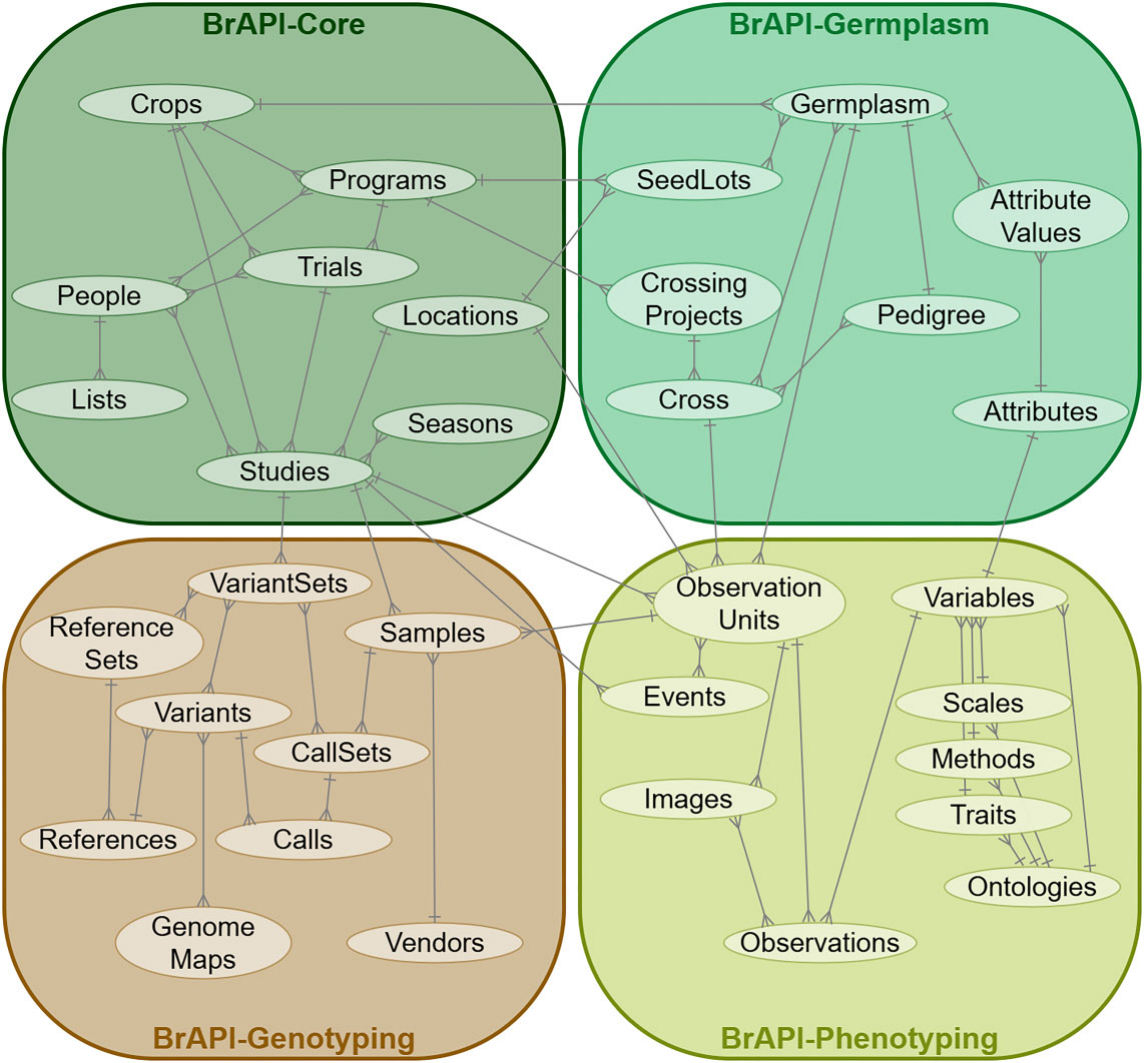
A simplified domain map of the whole BrAPI data model, divided into organizational modules. A more detailed Entity Relationship Diagram (ERD) is available on brapi.org.

Over its lifetime, the BrAPI project has grown and changed substantially. The total size of the specification has almost quadrupled since the release of version v1.0 in 2017, increasing from 51 endpoints in v1.0 to 201 endpoints in v2.1. Because of this growth, the specification documents were reorganized into four modules: BrAPI-Core, BrAPI-Phenotyping, BrAPI-Genotyping, and BrAPI-Germplasm. Figure 1 is a simplified domain map of the current BrAPI data model, showing what kinds of data are defined in each module.

The BrAPI specification follows a simple versioning scheme which attempts to balance the static nature of a standard with the ability to fix issues and innovate over time. The basic strategy involves major version and minor version updates. Minor versions are always made backwards compatible within a major version. Major versions are not guaranteed to be backwards compatible and usually come with sweeping structural changes to the whole specification. For example, a version v1.1 client application should still work with a version v1.3 server, but is not guaranteed to work with a v2.0 server. Each new version of the specification is built almost entirely from community suggestions, and the whole community has an opportunity to review a new version before it is officially released. This ensures that specification enhancements are driven by real use cases and the needs of the community.

The major version update from v1.3 to v2.0 allowed for the restructuring into the modules described in Figure 1, as well as a number of critical enhancements. While the v1.X versions of the specification focused on read-only phenotype data, the specification now has representation from most of the major concepts related to breeding and allows for read, write, and update capabilities. The v2.0 specification was updated to be more internally consistent, easier to navigate, and to provide more robust search capabilities. Major updates were also made to align BrAPI with some of the other relevant breeding data standards, including the GA4GH Variants Schema [4] and the Minimal Information About a Plant Phenotyping Experiment (MIAPPE) guidelines [5]. This addition ensures comprehensive and standardized documentation of experimental metadata to improve data interoperability and reuse.

As BrAPI has matured, so have the tools, services, and libraries that work with the specification. Each new version is released with a change log to guide developers as they upgrade, an Entity Relationship Diagram (ERD) to visually describe the data model, and a JSON Schema data model to be used for automated development efforts. For groups using Java, JavaScript, Python, R, or Drupal, community-maintained libraries are available with full BrAPI implementations ready to be integrated into existing code. The BrAPI Test Server is updated to support every version of the specification for testing purposes. Finally, there are resource pages on the project website that showcase BrAPI-compatible applications and data resources available in the community.

### Community growth

The international BrAPI Community consists of software developers, biologists, and other scientists working on BrAPI-related projects and data sources. This community sustains the BrAPI project, builds implementations, maintains development tools, and provides input to enhance the specification. As the project has grown, so too has the community. The BrAPI project started in June 2014 with fewer than ten people coming together to discuss the idea and has since grown to more than 200 members.

The BrAPI Hackathons are a major staple of the BrAPI community [6]. Twice a year, the community gathers in person or virtually to discuss the specification and collaborate on BrAPI-related projects. These events have proven to be vital to the long-term growth of the community; for some organizations, the hackathon is the only time during the year when they can collaboratively work on BrAPI projects.

## Results

**Figure 2.**
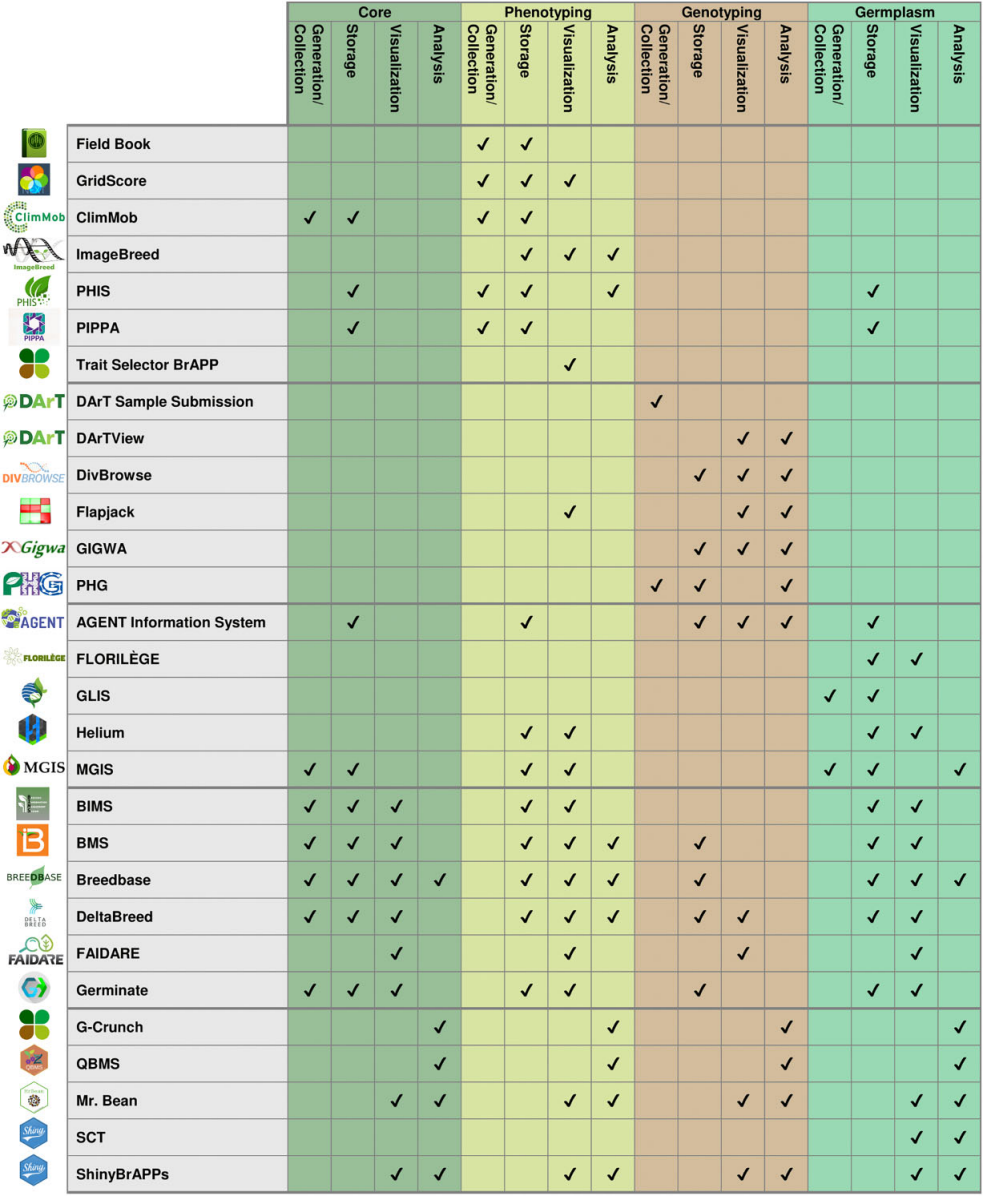
A summary of all the tools described below and the general areas each tool is designed to handle. The ‘Generation/Collection’ column indicates that an application is used to input or create new data. The ‘Storage’ column indicates the tool stores that type of data. The ‘Visualization’ column indicates that the application has a way of presenting data to a human user. The ‘Analysis’ column indicates the tool performs calculations to provide new insight.

Below, we present a number of short success stories from the BrAPI community. These tools, applications, and infrastructure projects serve as another indicator of community growth and success over the past 5 years. The vast majority of these tools have been developed and maintained by organizations independent of the BrAPI project, further demonstrating the impact of the project. These stories clearly illustrate all the different ways the BrAPI standard can be used productively and in practice. Figure 2 contains a summary of many of the currently available BrAPI-compliant tools, and each will be further described below. The list presented here showcases the breadth of BrAPI capabilities, but it is not an exhaustive list of BrAPI-compliant tools. A more comprehensive list can be found on the Compatible Software page of the BrAPI project website.

### Phenotyping

Phenotyping is fundamental to plant breeding and genetics research, providing the data breeders rely on to make informed selection decisions. Effective phenotyping requires a strong biological framework and robust data collection methods to ensure successful outcomes. The BrAPI specification supports phenotypic data throughout their lifecycle, including collection, analyses, publication, and archiving. To facilitate standardized data management, the BrAPI community has developed several BrAPI-compatible tools that streamline data curation, storage, and metadata integration. Ongoing development efforts are creating tools to manage images and other high-throughput phenotypic data sources, further enhancing the precision and efficiency of breeding research. By enabling the seamless transfer of phenotypic data, BrAPI-compatible tools simplify the conversion of raw observations into actionable insights, accelerating the digitization of modern breeding and genetics research programmes. The following set of BrAPI-compatible tools were developed to support various aspects of the phenotyping process.

#### Field book

Data from plant breeding and genetics experiments has traditionally been collected using pen and paper, but this approach often results in transcription errors and delayed analysis. Field Book [7], a highly customizable Android app, was developed to help scientists digitize and organize their phenotypic data as measurements and images are collected. This effectively improves data collection speed, reduces errors, and enables larger and more robust breeding populations and data sets.

Field Book has added support for BrAPI to streamline data transfer to and from BrAPI-compatible servers. This improvement has removed the need to manually transfer data files, simplifies data exchange between these systems, and reduces the opportunities for human error and data loss.

#### GridScore

GridScore [8] is a web-based application for recording phenotypic observations that harnesses mobile devices to enrich the data collection process. The GridScore interface closely mirrors the look and feel of printed field plans, creating an intuitive user experience. GridScore performs a wide range of functions, including data validation, data visualization, georeferencing, multiplatform support, and data synchronization across multiple devices. The application’s data collection approach employs a top-down view onto the trial and offers field navigation mechanisms using barcodes, QR codes, or guided walks that take the data collector through the field in one of 16 predefined orders.

BrAPI has further increased the value of GridScore by integrating it into the overarching plant breeding workflow. Trial designs and trait definitions can be imported into GridScore using BrAPI, and a finalized trial can be exported via BrAPI to any compatible database.

#### ClimMob

ClimMob [9] is a software suite designed to facilitate decentralized agricultural research through citizen science and choice experiments. It enables large-scale participatory trials where farmers and other stakeholders evaluate and rank different crop accessions or agronomic practices based on their preferences and field performance. While these data may lack the detail of centralized experiments, they can provide robust evidence on performance across a wide range of environments with increased external validity. Applications of ClimMob include crop variety testing, evaluating agronomic practices, and investigating climate resilience strategies. The platform supports experimental design, data collection through mobile apps, and data analysis to provide actionable insights, making on-farm trials accessible to farmers, breeders, and other stakeholders.

ClimMob uses BrAPI to retrieve curated germplasm information from breeding databases for trial design, subsequently enabling the automatic upload of ClimMob-collected data to a central breeding database for long-term storage and analysis. Analysed data can also be pushed from ClimMob to breeding databases, providing breeders with insights into the potential adoption of the tested crop varieties.

#### ImageBreed

Unoccupied aerial and ground vehicles (UAVs and UGVs) enable the high throughput collection of images and other sensor data in the field, but the rapid processing and management of these datasets are often a bottleneck for breeding programmes seeking to deploy these technologies for time-sensitive decision-making. ImageBreed [10] is an open-source, BrAPI-compliant image processing tool that supports the routine use of UAVs and UGVs in breeding programmes through standardized pipelines. It creates orthophotomosaics, applies filters, assigns plot polygons, and extracts ontology-based phenotypes from raw UAV-collected images. The BrAPI standard is used to push these phenotypes back to a central BrAPI-compliant breeding database where they can be analysed with other experiment data. The ImageBreed team has collaborated with others in the community to enhance the BrAPI image data standards, which it uses to upload raw images to a central breeding database or any other BrAPI-compatible long-term storage service.

#### PHIS

PHIS [11], the Hybrid Phenotyping Information System, is an ontology-driven information system based on semantic web technologies and the OpenSILEX framework. PHIS is deployed in several field and greenhouse platforms of the French national PHENOME and European EMPHASIS infrastructures. It manages and collects data from basic phenotyping and high-throughput phenotyping experiments on a daily basis. PHIS unambiguously identifies the objects and traits in an experiment and establishes their types and relationships via ontologies and semantics.

Since its inception, PHIS has been designed to be BrAPI compliant, encompassing the Core, Phenotyping, and Germplasm BrAPI modules. This enables integration with other BrAPI-compliant systems and platforms, simplifying the exchange of accession and phenotyping data across systems. PHIS is actively integrated with the OLGA genebank accessions management system and is indexed by the FAIDARE data portal [12]. BrAPI-enabled interoperability promotes a more coherent and efficient approach to the management and use of phenotyping data on various platforms and research initiatives within the European scientific community. BrAPI compliance also ensures that PHIS is compatible with other standards such as MIAPPE [5]. By integrating BrAPI requirements into its structure, PHIS strengthens its capacity for interoperability and effective collaboration in the wider context of plant breeding and related fields.

#### PIPPA

PIPPA [13] is a data management system used for collecting data from the Weighing, Imaging, and Watering Machines (WIWAM) [14], which are a range of automated high-throughput phenotyping platforms. These platforms have been deployed by research institutes and commercial breeders across Europe. They can be set up in a variety of configurations with different types of equipment, including weighing scales, cameras, and environment sensors. The software features a web interface with functionality for setting up new experiments, planning imaging and irrigation treatments, linking metadata (genotype, growth media, manual treatments) to pots, and importing, exporting, and visualizing data. It also supports the integration of image analysis scripts and connects to a compute cluster for job submission.

To share the phenotypic data from PIPPA experiments linked to publications, an implementation of BrAPI v1.3 was developed which allowed read-only access to the data in the BrAPI standardized format. This server was registered on FAIDARE, allowing the data to be found alongside data from other BrAPI-compatible repositories.

Throughout its development, the PIPPA project has adhered to guidelines set forth by BrAPI and the MIAPPE scientific standard. Current efforts are focused on delivering a public BrAPI v2.1 endpoint and increasing the availability of public high-throughput datasets via BrAPI.

#### Trait selector BrAPP

The Trait Selector BrAPP is a JavaScript-based application used to visually search and select traits from an ontology. The Trait Selector employs a visual aid, an image of a plant, to connect plant anatomy with relevant trait ontology terms. Instead of scrolling through a long list of possible traits, the user can click on pieces of the image to show the traits associated with specific plant structures. The Trait Selector BrAPP can be used to quickly find specific traits or to identify accessions that have a specific phenotype of interest.

The Trait Selector BrAPP has been successfully added to Cassavabase [15] and MGIS [16], and it can be integrated into any website or system with a BrAPI-compatible data source. A breeding database would need to only implement the BrAPI endpoints for Traits, Observations, and Variables, while a genebank would require only Traits and Germplasm Attributes.

### Genotyping

Genotyping has become a cornerstone of most breeding processes, but managing the data can be challenging. The choice of genotyping platform depends largely on the crop species, research objectives, and available resources. Techniques such as single nucleotide polymorphism (SNP) genotyping, genotyping-by-sequencing (GBS), simple sequence repeats (SSRs), whole genome sequencing (WGS), and array-based genotyping each offer specific advantages depending on the crop and research objectives. BrAPI supports genotypic data by utilizing existing standards such as the variant call format (VCF) [17] and the GA4GH Variants schema [4]. The BrAPI community has developed compatible tools for storing, searching, visualizing, and analysing genotypic data, making it easier to integrate and utilize this information in breeding programmes. These BrAPI-compliant tools streamline data management and analysis, enhancing the breeders’ ability to make data-driven decisions in developing superior crop varieties.

#### DArT sample submission

The Diversity Arrays Technology (DArT) genotyping lab is heavily used worldwide for plant genotyping. With over 1200 available organisms and species, a client base on every continent, and many millions of samples processed, DArT provides services for several generic and bespoke genotyping technologies and solutions. Processes of sample tracking and fast data delivery are at the core of the ordering system developed at DArT. The ordering system is tightly integrated with DArTdb (DArT’s custom LIMS operational system), which drives laboratory, quality, and analytical processes.

DArT has been a part of the BrAPI community since its inception. DArT developers have worked with the BrAPI community, contributing to various aspects of the API specification. One key aspect was establishing a standard API for sending sample metadata to the lab for genotyping. This solution eliminates much of the human error involved with sending samples to an external lab and allows for an automated process of sample batch transfers. The current implementation also allows for an order status verification, automated data discovery, and data downloads. Data are delivered as standard data packages with self-describing metadata.

The current BrAPI implementation at DArT is in production, and it is compatible with the BrAPI v2.1 specification. Further details about DArT’s ordering system can be found at DArT Ordering System and also at DArT Help.

#### DArTView

DArTView is a desktop application for marker data curation via metadata filtering. DArTView enables genotype variant data visualization designed such that users can easily identify trends or correlations within their data. The primary goal of the tool is to overcome tedious manual calculation of marker data through common spreadsheet applications like Excel. Users are able to import marker data from CSV files, but DArTView has been recently enhanced to be BrAPI compatible. BrAPI provides a consistent data standard across databases and data resources, which allows DArTView to use any BrAPI-compatible server as an input data source. DArTView’s compatibility with BrAPI also ensures easy integration with other tools and pipelines that would use DArTView for marker filtering and exploration.

Initially developed by DArT, the tool is gaining popularity within the breeding community, especially in Africa. Future releases will focus on enhancing the BrAPI compatibility, making it accessible to more breeders and researchers. A web-enabled version of DArTView is in development. This new version will allow for further collaboration opportunities with other interested partners who would like to integrate it as part of their pipelines.

#### DivBrowse

DivBrowse [18] is a web platform for exploratory data analysis of large genotyping studies. The software can be run standalone or integrated as a plugin into existing web portals. At its core, DivBrowse combines the convenience of a genome browser with features tailored to germplasm diversity analysis. DivBrowse provides visual access to VCF files obtained through genotyping experiments and can handle hundreds of millions of variants across thousands of samples. It is able to display genomic features such as nucleotide sequence, associated gene models, and short genomic variants. DivBrowse also calculates and displays variant statistics such as minor allele frequencies, the proportion of heterozygous calls, and the proportion of missing variant calls. Dynamic PCAs can be performed on a user-specified genomic area to provide information on local genomic diversity. DivBrowse has an interface to BLAST+ tools [19] installed on Galaxy servers [20], which can be used to directly access genes or other genomic features from the results of custom BLAST queries. DivBrowse employs the BrAPI-Genotyping module to serve genotypic data as a BrAPI endpoint and to get genotypic data from other BrAPI endpoints.

#### Flapjack

Flapjack [21] is a multiplatform desktop application for data visualization and breeding analysis (e.g. pedigree verification, marker-assisted backcrossing and forward breeding) using high-throughput genotype data. Data can be imported into Flapjack from any BrAPI-compatible data source with genotype data available. Flapjack Bytes is a smaller, lightweight, and fully web-based counterpart to Flapjack that can be easily embedded into a database website to provide similar visualizations online. Traditionally supporting its own text-based data formats, Flapjack’s use of BrAPI has streamlined the end-user experience for data import. Work is underway to determine the best methods to exchange analysis results using future versions of the API.

#### Gigwa

Gigwa is a Java EE web application that provides a means to centralize, share, finely filter, and visualize high-throughput genotyping data [22]. Built on top of MongoDB, it is scalable and can support working smoothly with datasets containing billions of genotypes. It is installable as a Docker image or as an all-in-one bundle archive. It is straightforward to deploy on servers or local computers and has thus been adopted by numerous research institutes from around the world. Notably, Gigwa serves as a collaborative management tool and a portal for exploring public data for genebanks and breeding programmes at some CGIAR centres [23]. The total amount of data hosted and made widely accessible using this system has continued to grow over the last few years.

The Gigwa development team has been involved in the BrAPI community since 2016 and took part in designing the genotype-related section of the BrAPI standard. Gigwa’s first BrAPI-compliant features were designed for compatibility with the Flapjack visualization tool [21]. Over time, Gigwa has established itself as the first and most reliable implementation of the BrAPI-Genotyping module. Local collaborators and external partners used it as a reference solution to design a number of tools taking advantage of the BrAPI-Genotyping features (e.g. BeegMac, SnpClust, QBMS).

Some use-cases require Gigwa to also consume data from other BrAPI servers. This requirement led to the implementation of BrAPI client features within Gigwa. A close collaboration was established with the Integrated Breeding Platform (IBP) team and their widely used breeding management system (BMS). This collaboration means both applications are now frequently deployed together; Gigwa pulling germplasm or sample metadata from BMS, and BMS displaying Gigwa-hosted genotypes within its own UI.

#### PHG

The Practical Haplotype Graph (PHG) is a graph-based computational framework that represents large-scale genetic variation and is optimized for plant breeding and genetics [24]. Using a pangenome approach, each PHG stores haplotypes (the sequence of part of an individual chromosome) to represent the collective genes of a species. This allows for a simplified approach for dealing with large-scale variation in plant genomes. The PHG pipeline provides support for a range of genomic analyses and allows for the use of graph data to impute complete genomes from low-density sequence or variant data.

Users can access the haplotype data either with direct calls to the PHG embedded server or indirectly using the rPHG library from an R environment. The PHG server accepts BrAPI queries to return information on sample lists and the variants used to define the graph’s haplotypes. In addition, PHG users utilize the BrAPI variant sets endpoint query to return links to VCF files containing haplotype data. Work on the PHG is ongoing, and it is expected to support additional BrAPI endpoints that allow for fine-tuned slicing genotypic data in the near future.

### Germplasm management

Germplasm data management is essential for research or breeding programmes, national genebanks, and international collaborations. A substantial number of new accessions, variants and lines are developed every year and preserved in collections around the world. Germplasm are an important source of biological diversity for breeders to further develop crops. To support these efforts, BrAPI allows for the exchange of germplasm passport data, pedigree trees, and crossing metadata. The BrAPI community has developed compliant tools for storing, searching, and visualizing this metadata, facilitating efficient management. Additionally, there are plans to establish federated networks of genebank data connected via BrAPI, enhancing global accessibility and collaboration in germplasm management.

#### AGENT

The aim of the AGENT project, funded by the European Commission, is to develop a concept for the digital exploitation and activation of plant genetic resources (PGRs) throughout Europe [25]. In the global system for *ex situ* conservation of PGRs, material is being conserved in about 1750 collections totalling ∼5.8 million accessions [26]. Unique and permanent identifiers in the form of DOIs are available for more than 1.9 million accessions via the Global Information System (GLIS) [27] of the International Treaty on Plant Genetic Resources for Food and Agriculture (ITPGRFA). Each DOI is linked to some basic descriptive data that facilitates the use of these resources, mainly passport data. However, a data space beyond the most basic information is needed that includes genotypic and phenotypic data. This space will help to answer questions about the global biological diversity of plant species, the detection of duplicates, the tracking of provenance for the identification of genetic integrity, the selection of the most suitable material for different purposes, and to support further applications in the field of data mining or AI. In this context, the AGENT project activates and utilizes the PGRs from European *ex situ* genebanks according to the FAIR principles [28] and tests the resources in practice using two important crops, barley and wheat. Thirteen European genebanks and five bioinformatics centres are working together and have agreed on standards and protocols for data flow and data formats for the collection, integration, and archiving of genotypic and phenotypic data [29].

The BrAPI specification is one of the agreed standards that are detailed in the AGENT guidelines for dataflow [30]. The implemented BrAPI interface enables the analysis of current and historic genotypic and phenotypic information. This will drive the discovery of genes, traits, and knowledge for future missions, complement existing information for wheat and barley, and use the new data standards and infrastructure to promote better access and use of PGR for other crops in European genebanks. The AGENT database backend aggregates curated passport data, phenotypic data, and genotypic data on wheat and barley accessions of 18 project partners. This data is accessible via BrAPI endpoints and explorable in a web portal. Genotyping data uses the DivBrowse [18] storage engine and its BrAPI interface. Soon, the BrAPI implementation will be expanded to enable the integration of analysis pipelines in the AGENT infrastructure, such as the FIGS + pipeline developed by ICARDA [31]. In addition, the data collected by the AGENT project will be integrated into the European Search Catalogue for Plant Genetic Resources (EURISCO) [32].

#### Florilège

Florilège is a web portal designed primarily for the general public to access public PGRs held by the Biological Resource Centers across France, as part of France’s National Research Institute for Agriculture, Food and Environment (INRAE). Through this portal, users can browse accessions from over 50 plant genera spread across 19 genebanks. It allows users to view available seeds and plant material, including options for ordering material. Florilège provides centralized access to the various French collections of PGRs available to the public.

Florilège retrieves accession information from several BrAPI-compliant systems. Key among these are OLGA, a genebank accessions management system, and GnpIS, an INRAE data repository for PGRs, phenomics, and genetics [33, 34]. Using BrAPI to gather data from these systems reduced development efforts and enabled standardized data retrieval. As a result, BrAPI has become the de facto standard within the French PGRs community for exchanging information. During development, the Florilège team also proposed several enhancements to the BrAPI specifications themselves, such as additional support for Collection objects or improved reference linking, to better accommodate their specific use case.

#### GLIS

The GLIS on Plant Genetic Resources for Food and Agriculture (PGRFA) of the International Treaty on PGRFA (ITPGRFA) is a web-based, BrAPI-compliant global entry point for PGRFA data [27]. It allows users and third-party systems to access information and knowledge on scientific, technical, and environmental matters to strengthen PGRFA conservation, management, and utilization activities. The system and its portal also enable recipients of PGRFA to make available all non-confidential information on germplasm according to the provisions of the Treaty and facilitate access to the results of their research and development.

Thanks to the adoption of Digital Object Identifiers (DOIs) for Multi-Crop Passport Descriptors (MCPD) of PGRFA accessions, the GLIS Portal provides access to 1.9 million PGRFA in collections conserved worldwide. Of these, over 1.5 million are accessible for research, training and plant breeding in the food and agriculture domain.

The Scientific Advisory Committee of the ITPGRFA has repeatedly welcomed efforts on interoperability among germplasm information systems. In this context, the GLIS Portal adopted the BrAPI v1.3 in 2022. Integrating BrAPI among the GLIS content negotiators facilitates queries and the exchange of content for data management in plant breeding. The Portal also offers other protocols (XML, DarwinCore, JSON and JSON-LD) to increase data and metadata connectivity. In the near future, depending on the availability of resources, upgrading to BrAPI v2 is planned.

#### Helium

Helium [35] is a pedigree visualization platform designed to account for the unique characteristics of plant pedigrees. A pedigree is a representation of the genetic relationships between discrete individuals, linking individual plants or lines with their parents and progeny. Plant pedigrees inform crossing decisions, are required for variety releases, and are often used to check for potential genotyping or phenotyping errors [36]. The accurate representation of pedigrees and the ability to pull pedigree data from different data sources are important in plant breeding and genetics. Helium provides ways to visualize and interact with this complex data in meaningful ways.

From its original desktop interface, Helium has developed into a web-based visualization platform implementing BrAPI calls to allow users to import data from other BrAPI-compliant databases. The ability to pull data from BrAPI-compliant data sources has significantly expanded Helium’s capability and utility within the community. Helium is used in projects ranging in size from tens to tens of thousands of lines and across a wide variety of crops and species. While originally designed for plant data [37], it has also found utility in other non-plant projects [38] highlighting its broad utility. BrAPI also allows Helium to provide direct dataset links to collaborators, allowing the original data to be held with the data provider and utilizing Helium for its visualization functionality. Our current Helium deployment includes example BrAPI calls to a barley dataset at the James Hutton Institute to allow users to test the system and features it offers.

#### MGIS

The Musa Germplasm Information System (MGIS) serves as a comprehensive community portal dedicated to banana diversity, a crop critical to global food security [16]. MGIS offers detailed information on banana germplasm, focusing on the collections held by the CGIAR International Banana Genebank (ITC) [39]. It is built on the Drupal/Tripal technology, like BIMS [40] and Florilège.

Since its inception, MGIS developers have actively participated in the BrAPI community. The MGIS team pushed for the integration of the MCPD standard into the Germplasm module of the API. MCPD support was added in BrAPI v1.3, and MGIS now provides passport data information on ITC banana genebank accessions (with GLIS DOI), synchronized with Genesys. MGIS also enriches the passport data by incorporating additional information from other germplasm collections worldwide. All the germplasm data is available through the BrAPI-Germplasm module implementation. For genotyping data, MGIS integrates with Gigwa [22], which provides a tailored implementation of the BrAPI genotyping module. Furthermore, MGIS supports a set of BrAPI-Phenotyping module endpoints, facilitating the exposure of morphological descriptors and trait information supported by ontologies like the Crop Ontology [41]. MGIS has integrated the Trait Selector BrAPP, and there are use cases implemented to interlink genebank and breeding data between MGIS and the breeding database MusaBase.

### Breeding and genetics data management

While specialty data management is important for some use cases, often breeders want a central repository or access point of critical data. General breeding and genetics data management systems and web portals support some level of phenotypic, genotypic, and germplasm data, as well as trial, equipment, and people management. By enabling BrAPI support, these larger systems can connect with smaller tools and specialty systems to provide more functionality under the same UI. There are several breeding data management systems developed in the BrAPI community, each with their own strengths.

#### BIMS

The Breeding Information Management System (BIMS) [40] is a free, secure, online BMS, which allows breeders to store, manage, archive, and analyse their private breeding programme data. BIMS enables individual breeders to have complete control of their own breeding data along with access to tools such as data import, export, analysis, and archiving for their germplasm, phenotype, genotype, and image data. BIMS is currently implemented in five community databases, the Genome Database for Rosaceae [42], CottonGEN [43], the Citrus Genome Database, the Pulse Crop Database, and the Genome Database for Vaccinium, where it enables individual breeders to import publicly available data. BIMS is also implemented in the public database breedwithbims.org that any breeder can use.

BIMS primarily utilizes BrAPI to connect with Field Book [7], enabling seamless data transfer between data collection and subsequent management in BIMS. BIMS can receive data from BreedBase [15] via BrAPI as well, and data transfer between BIMS and other resources such as GIGWA [22], and the Breeder Genomics Hub [44] is under development.

#### BMS

The BMS, developed by the IBP, is a suite of tools designed to enhance the efficiency and effectiveness of plant breeding. BMS covers all stages of the breeding process, with the emphasis on germplasm management and ontology-harmonized phenotyping (i.e. with the Crop Ontology). It also features analytics and decision-support tools. With its focus on interoperability, BMS integrates smoothly with BrAPI, facilitating easy connections with a broad array of complementary tools and databases. Notably, the BMS is often deployed together with Gigwa to fulfil the genotyping data management needs of BMS users.

The brapi-sync tool, a significant component of BMS’s BrAPI capabilities, was developed by the IBP and released as a BrAPP for community use. Brapi-sync is designed to enhance collaboration among partner institutes within a network such as Innovation and Plant Breeding in West Africa (IAVAO). The tool enables the sharing of germplasm and trial metadata across BrAPI-enabled systems. It helps overcome traditional barriers to collaboration, ensuring data that was once isolated within specific programmes or platforms can now be easily shared, integrated, and synchronized.

Additionally, Brapi-sync improves data management by maintaining links to the original source of each entity it transmits. This retains the original context of the data and establishes a traceability mechanism for accurate data source attribution and verification. Such practices are crucial for maintaining data integrity and fostering trust among collaborative partners, ensuring access to accurate, reliable, and current information.

#### Breedbase

Breedbase is a comprehensive, open-source breeding data management system [15,45] that implements a digital ecosystem for all breeding data, including trial data, phenotypic data, and genotypic data. Data acquisition is supported through data collection apps such as Fieldbook [7], Coordinate, and InterCross, as well as through drone imagery, Near Infra-Red Spectroscopy (NIRS), and other technologies. Search functions, such as the Search Wizard interface, provide powerful query capabilities. Various breeding-centric analysis tools are available, including mixed models, heritability, stability, principal component analysis (PCA), and various clustering algorithms. The original impetus for creating Breedbase was the advent of new breeding paradigms based on genomic information, such as genomic prediction algorithms [46] and the accompanying data management challenges. Thus, a complete genomic prediction workflow is integrated into the system.

The BrAPI interface is crucial for Breedbase. Breedbase uses BrAPI to connect with the data collection apps, other projects such as ClimMob [9], and native BrAPPs built into the Breedbase webpage. Users also appreciate the ability to connect to Breedbase instances using packages such as QBMS [47] for data import into R for custom analyses. The Breedbase team has been part of the BrAPI community since its inception and has continuously adopted and contributed to the BrAPI standard.

#### DeltaBreed

DeltaBreed is an open-source breeding data management system designed and developed by Breeding Insight to support U.S. Department of Agriculture—Agricultural Research Service (USDA-ARS) speciality crop and animal breeders. DeltaBreed differs from other related systems in that it is customizable to small breeding teams and generalized enough to support the workflows of diverse species. DeltaBreed is a unified system that integrates BrAPI applications, like the BrAPI Java Test Server, Gigwa, and the Pedigree Viewer BrAPP, through a common UI. BrAPI integration shields users from multifactorial differences existing between various applications. DeltaBreed, adhering to the BrAPI model, establishes data standards and validations for users and provides a singular framework for data management and user training. BrAPI also enables DeltaBreed users to connect to external tools with separate UIs, like Field Book, QBMS, Mr Bean, BreedBase, and Helium.

DeltaBreed users need not be aware of BrAPI specifics but will notice that BrAPI interoperability reduces the need for human-mediated file transfers and data manipulation. Field Book users, for example, can connect to their DeltaBreed program, authenticate, and pull studies and observation variables directly from DeltaBreed to Field Book on their data collection device. The subsequent step of pushing observations from Field Book to DeltaBreed is straightforward via BrAPI but is pending implementation until data quality validations are put in place; these include improved data transaction handling and differentiation of intentional and inadvertent repeated measures.

#### FAIDARE

FAIDARE [48] is a data discovery portal providing a biologist-friendly search system over a global federation of 40 plant research databases. It allows users to identify data resources using a full-text search approach combined with domain-specific filters. Each search result contains a link back to the original database for visualization, analysis, and download. The indexed data types are broad and include genomic features, selected bibliography, QTL, markers, genetic variation studies, phenomic studies, and PGRs. This inclusiveness is achieved thanks to a two-stage indexation data model. The first index, more generic, provides basic search functionalities and relies on five fields: name, link back URL, data type, species, and exhaustive description. To provide more advanced filtering, the second-stage indexation mechanism takes advantage of BrAPI endpoints to get more detailed metadata on germplasm, genotyping studies and phenotyping studies.

The FAIDARE indexation mechanism relies on a public software package [49] that allows data resource managers to request the indexation of their database. This BrAPI client is currently able to extract data from any BrAPI v1.3 and v1.2 endpoint, and the development of BrAPI v2.x indexation will be initiated in 2025. Since not all databases are willing to implement BrAPI endpoints, it is possible to generate metadata as static BrAPI-compliant JSON files, using the BrAPI standard as a file exchange format.

The FAIDARE architecture has been designed by elaborating on the BrAPI data model in combination with the GnpIS Software Architecture [33]. It uses an Elasticsearch NoSQL engine that searches and serves enriched versions of the BrAPI JSON data model. FAIDARE also includes a BrAPI endpoint using all indexed metadata. It has been adopted by several communities, including the ELIXIR and EMPHASIS European infrastructures, and the WheatIS of the Wheat Initiative. Several databases are added each year to the FAIDARE global federation, adding to both the portal and BrAPI adoption.

#### Germinate

Germinate [50,51] is an open-source PGRs database that combines and integrates various types of plant breeding data including genotypic, phenotypic, passport, image, geographic, and climate data into a single repository. Germinate is tightly linked to the BrAPI specification and supports the majority of BrAPI endpoints for querying, filtering, and submission.

Germinate connects with other BrAPI-enabled tools such as GridScore for phenotypic data collection, Flapjack for genotypic data visualization, and Helium for pedigree visualization. Additionally, due to the nature of BrAPI, Germinate can act as a data repository for any BrAPI-compatible tool. The interoperability provided by BrAPI reduces the need for manual data handling, providing the direct benefits of faster data processing, fewer human errors, and improved data security and integrity.

### Analytics

Modern breeding programmes have multiple decision points requiring analysis and integration of various data types. While there are numerous breeding and genetics data management systems (above), certain programme tasks could be simplified by the development of specific streamlined analysis systems. These analysis systems better enable certain tasks by utilizing data from different sources to make efficient data-driven decisions. With increased computational power at their disposal, scientists can construct more advanced analysis pipelines by combining various data sources.

The tools developed by the BrAPI community can pull in data from multiple BrAPI-compatible data sources and provide enhanced analytical functionality. In many cases, there is no longer a need to import and export large data files to a local computational environment just to run standard analytical models. These tools are able to extract the data they need from various data sources without much human intervention or human error. They can also provide simple user interaction to enhance decision support for the breeders and researchers.

#### G-Crunch

G-Crunch is an upcoming user-facing tool to make simple, repeatable analysis requests. The lightweight UI can be used to specify and filter incoming data, select specific analysis criteria, and trigger any analytics pipeline that is built into the specific framework instance. G-Crunch is currently built on top of the open-source Analytics Framework project and can run pipelines using tools such as Sommer and ASREML. Each piece of the data and pipeline can be separately specified, which can allow flexibility when running complex analysis. A ‘test’ analysis can be run on small data sets with a small or local analytics engine, then quickly redirect G-Crunch to a larger dataset and a larger computational framework. This mitigates the complications of moving data around and introducing errors from manually triggering the analysis steps.

G-Crunch relies on BrAPI endpoints to access phenotypic and genotypic data sources, as well as an API currently implemented in the Analytics Framework to start and track processes. G-Crunch, as a tool, could not feasibly exist without BrAPI. The support of BrAPI interfaces allows G-Crunch to use one unified request method and adapt to the user’s existing network of BrAPI-compliant tools. This lowers the barrier to entry for adoption and makes analysis pipelines easily repeatable.

#### QBMS

Many plant breeders and geneticists analyse their datasets using the R statistical programming language, but this requires the import of data into an R environment. BrAPI enables access to pull datasets into R from compatible databases, but API backend processes, such as authentication, tokens, TCP/IP protocol, JSON format, pagination, stateless calls, asynchronous communication, and database IDs, are complex for users to navigate. The QBMS R package eliminates technical barriers scientists experience when using the BrAPI specification in their analysis scripts and pipelines by providing breeders with stateful functions familiar to them when navigating their GUI systems [47]. QBMS enables users to query and extract data into a dataframe, a common structure in the R language, providing an intuitive connection with breeding data management systems.

The community has built extended solutions on top of QBMS, incorporating the package into R-Shiny BrAPPs such as MrBean [52] (described below). QBMS is open-source and available on the official CRAN repository, where it has garnered over 16,000 downloads.

#### Mr.Bean

MrBean [52] is a GUI designed to assist breeders, statisticians, and individuals involved in plant breeding programmes with the analysis of field trials. By utilizing innovative methodologies such as SpATS for modelling spatial trends and autocorrelation models to address spatial variability, MrBean proves highly practical and powerful in facilitating faster and more effective decision-making. Modelling genotype-by-environment interaction poses its challenges, but MrBean offers the capability to explore various variance-covariance matrices, including factor analytic, compound symmetry, and heterogeneous variances. This aids in the assessment of genotype performance across diverse environments.

MrBean boasts flexibility in importing different file types, yet for users managing their data within data management systems, the process of downloading from their systems and importing it into MrBean can be cumbersome. To address this issue, QBMS was integrated into the back end. This feature prompts users to input the URL of a BrAPI-compatible server, enter their credentials (if necessary), and select the specific trial they wish to analyse. Subsequently, users can seamlessly access their dataset through BrAPI and utilize it across the entire MrBean interface.

#### SCT

The Sugarcane Crossing Tool (SCT) is a lightweight R-Shiny dashboard application designed to receive, process, and visualize data from a linked BreedBase [15] instance. This application is being developed collaboratively with members of the Sugarcane Integrated Breeding System, who have advocated for an application that assists them in designing crosses based on queried information from a list of available accessions. By leveraging existing community resources, the team has been able to develop a simple, BrAPI-enabled application without possessing extensive programming knowledge or experience. The SCT is presented as an inspiration for similarly positioned scientists to consider developing custom applications for specific tasks.

The crossing tool utilizes a modified version of the BrAPI-R library to access a compliant database, and it employs standard R/JavaScript packages to aggregate and visualize data. Modules within the application allow breeders to query the database (through BrAPI) for information relevant to their decision-making process. This includes the number and sex of flowering accessions, deep pedigree and relatedness information, summarized trial data, and the prior frequency and success of potential cross combinations. Future versions of this tool will provide additional decision support (e.g. ranked potential crosses) to enhance the accuracy and efficiency of crossing.

#### ShinyBrAPPs

The ShinyBrAPPs code repository contains a number of useful tools, built using the R-Shiny framework and the BrAPI-R open-source library. The R-Shiny framework allows user communities to quickly prototype and produce applications that are finely tailored to their needs, thus improving adoption and daily use of data management systems. An international collaboration of developers from CIRAD and the IBP have been working together as part of the IAVAO breeders community to develop these ShinyBrAPPs in support of national breeding programmes in western Africa. These applications are typically connected to BMS and/or Gigwa and provide tools for specific use cases. BrAPI compliance offers these systems the opportunity to add functionalities in a modular way through the development of external plugin applications that can quickly fulfil specific needs for this group of breeders and scientists.

So far, four applications have been developed covering the fields of trial data quality control, single trial statistical analysis, breeding decision support, and raw genotyping data visual inspection. The ‘TDxPLOR’ (trial data explorer) application retrieves data from multilocation trials and displays data counts and summary boxplots for all variables measured in different studies. It also provides an interactive distribution plot to easily select observations that require curation and a report of candidate issues that need to be addressed by the breeder. ‘STABrAPP’ is an application for single-trial mixed- model analysis. It basically provides a GUI to the Statgen-STA R package. The ‘BrAVISE’ application is a decision support tool helping breeders to run GxE analysis, select germplasm according to their various characteristics and save lists of selected germplasm to the BMS. Finally, the ‘snpclust’ tool enables a user to check and manually correct the clustering of fluorescence-based SNP genotyping data.

### General infrastructure

Adopting BrAPI compatibility into an existing system can be difficult sometimes. The BrAPI Community has developed several tools to make adoption easier, built to support other programmers and developers. This includes things like prebuilt code libraries, connectors to other technology standards, and mappers to alternate data types or data files. The goal is to lower the barrier to entry for the BrAPI community, making it easier for other groups to get started and connect their existing data to the standard.

#### BrAPIMapper

BrAPIMapper is a full BrAPI implementation designed to be a convenient wrapper for any breeding-related data source. BrAPIMapper is provided as a Docker application that can connect to a variety of external data sources, including MySQL or PostgreSQL databases, generic REST services, flat files (XML, JSON, CSV/TSV/GFF3/VCF, YAML), or any combination of these. It provides an administration UI to map BrAPI data models to external data sources. The interface allows administrators to select the BrAPI specification versions to use and which endpoints to enable. Data mapping configuration import and export features simplify upgrades to future BrAPI versions; administrators only have to map missing fields or make minor adjustments. BrAPIMapper supports the primary BrAPI features, including paging, deferred search results, user lists, and authentication. Access restrictions to specific endpoints can be managed through the administration interface as well. This tool aims to accelerate BrAPI services deployment while ensuring specification compliance.

#### MIRA and BrAPI2ISA

In some communities and projects, phenotyping data and metadata are archived and published using files in ISA formats and validated using the MIAPPE ISA configuration [53]. Although ISA-Tab is easy to read for non-technical experts due to its file-based approach, it lacks programmatic accessibility, particularly for web applications. MIRA is a tool developed by IPK and extended during community hackathons to address this challenge. MIRA acts as an intermediary between a web application and the ISA-formatted dataset by automating the deployment of a BrAPI v2.1 server in a Docker container. Leveraging the MIAPPE mapping between the ISA data model and the BrAPI data model, MIRA facilitates the integration of data and metadata from phenotyping experiments and makes the ISA data programmatically accessible. The BrAPI server implements all the BrAPI endpoints required to access MIAPPE-compliant data and metadata.

The BrAPI2ISA service is the counterpart to MIRA, functioning as a converter between a BrAPI-compatible server and the ISA-Tab format. The tool simplifies, automates, and facilitates the archiving of data, thereby enhancing data preservation and accessibility. The BrAPI2ISA tool is compatible with BrAPI v1.3 and welcomes community contributions to support the latest versions of BrAPI.

#### GraphQL data-warehouse

GraphQL is a newer web service API architecture that offers an alternative to REST by enabling clients to retrieve exactly the data they need through a single, flexible query. Whereas REST focuses on representing individual resources, GraphQL defines a network, or graph, of data entities. The BrAPI data model can be extracted from the original RESTful documentation and repurposed in a GraphQL system. Using the Zendro software generator, a fully functional, cloud-capable data warehouse can be created from the current version of the BrAPI data models. This generated warehouse offers similar functionalities through its GraphQL API that BrAPI offers, including secure access to create, read, update, and delete (CRUD) operations standardized across all BrAPI data models. Zendro supports many underlying database systems, offering flexibility during installation and integration.

The BrAPI GraphQL server is particularly rich in data search and discovery features. Logical filters allow for exhaustive search queries constructed as logical triplets consisting of a BrAPI model property, a logical operator, and a value (e.g. ‘studyName equals “Nursery Study”’). Searches can be extended over relationships between data models, thus enabling a user to traverse the data model graph and query the warehouse for exactly the required data. An example data warehouse is publicly available and offers full read access in the UI and through the GraphQL API. The example warehouse is populated with public CassavaBase data [15] to demonstrate BrAPI-compliant data examples based on real data. Three interactive scientific example plots are available to explore the data.

## Discussion

### BrAPI for breeders

While the BrAPI technical specification is designed to be read and used by software developers, its underlying purpose is to support the work of breeders and other scientists by making routine processes faster, easier, and cheaper. BrAPI offers a convenient path to automation, interoperability, and data integration for software tools in breeding, genetics, phenomics, and other related agricultural domains. By integrating the tools described above, breeders and scientists can spend less time on data management and more time focusing on science. For many, the ultimate goal is the development of a digital data ecosystem: a collection of software tools and applications that can all work together seamlessly. In this scenario, data is digitally collected, automatically sent to quality control systems, batch analysed to provide actionable insights, and finally stored in accessible databases for long-term applications. As tools continue to adopt the BrAPI standard, this vision is beginning to approach reality.

### Looking ahead

The BrAPI project leadership and community are committed to building standards to support new use cases and technologies as they are adopted by breeders and other scientists, potentially including drone imaging data, spectroscopy, LIDAR, metabolomics, transcriptomics, agronomics, high-throughput phenotyping, pangenomics, and machine learning-based analysis. Each of these technologies will have unique challenges, generate different types of data, and require substantial thought and discussion before being added to the BrAPI specification. This process has already begun for several data types, with small groups working to build generic data models and proposed communication standards. As these community efforts are completed, they will become part of a future version of the BrAPI standard, enabling further interoperability and simplifying data exchange.

Expanding the BrAPI specification is important for the community, but this growth should not reinvent or compete with existing functional standards. Additions to the BrAPI specification are reviewed thoroughly by the community to make sure BrAPI is compliant with existing standards and data structures. For example, the community has requested compliance with the GFF3 standard for genomic data and the GeoTIFF standard for aerial image data. Pieces of these existing popular data structures might be integrated into the overall BrAPI standard documentation. In some cases, BrAPI will only reference other standards instead of including them in the specification. For example, there have been community discussions around developing connections with the NOAA CDO standard for weather data or the Galaxy Analytics API for analytics pipeline controls and information. These standards are perfectly adequate on their own, and recreating them in the BrAPI standard would be redundant.

## Conclusion

The BrAPI project only exists because of the community of software engineers, biologists, and other scientists who support and use it. While there were many tools and use cases presented here, it is not an exhaustive list of all BrAPI-compliant systems. As long as the standard continues to be supported, the community will continue to expand. As more groups continue to make their tools BrAPI compliant, others will see the value in implementing BrAPI into their own tools, allowing the community to strengthen and grow. By providing an open standard for breeding data and the infrastructure and community to support it, the BrAPI project is doing its part to support a productive agricultural system amidst the pressing challenges of climate change. If this manuscript is your first introduction to the BrAPI project, the authors invite you to join the community. More information is available at brapi.org.

## Data Availability

No new data were generated or analysed in support of this research.
